# Rapid Cooling Delays the Occurring of Core Browning in Postharvest ‘Yali’ Pear at Advanced Maturity by Inhibiting Ethylene Metabolism

**DOI:** 10.3390/foods13071072

**Published:** 2024-03-31

**Authors:** Hongyan Zhang, Yunyun Han, Liya Liang, Bing Deng

**Affiliations:** 1 College of Food Science and Biological Engineering, Tianjin Agricultural University, Tianjin 300392, China; 18789420684@163.com (H.Z.); liangliya@126.com (L.L.); 2 College of Horticulture, Shanxi Agricultural University, Taigu 030801, China; hanyunyun_x@163.com; 3 College of Food Science and Engineering, Shanxi Agricultural University, Taigu 030801, China

**Keywords:** ‘Yali’ pear, core browning, rapid cooling, respiration intensity, ethylene, senescence

## Abstract

During the storage and transportation processes, the occurrence of core browning in ‘Yali’ pear fruit due to adversity injury can be easily mitigated by implementing different cooling methods, especially in advanced maturity fruits. In this study, ‘Yali’ pears at an advanced maturity stage were subjected to slow cooling and rapid cooling treatment. The quality-related physiological percentage and severity, and the rate of good fruits were determined, and RNA-seq was used to explore the effects of different cooling methods on pathways related to core browning in advanced-maturity pears at the transcriptional level. The results indicated that, compared with slow cooling treatment, rapid cooling significantly inhibited core browning in advanced-maturity ‘Yali’ pears. Measurements of quality-related physiological indexes suggested that rapid cooling treatment led to higher SSC content, firmness, L* value, and b* value, indicating better brightness, coloration, and higher soluble solid content, which are desirable for commercial sale. Rapid cooling effectively suppressed the physiological metabolism of ‘Yali’ pears, delaying fruit senescence compared with slow-cooling treatment. Furthermore, the RNA-Seq sequencing results revealed that pathways related to browning are involved in hormone signal transduction pathways, which are associated with resistance and aging processes of pear fruit. In summary, rapid cooling treatment delayed the core browning of advanced maturity of ‘Yali’ pears, indicating that the core browning of ‘Yali’ pears is related to the cooling method, and the mechanism of rapid cooling in reducing the core browning of advanced maturity of ‘Yali’ pears was by delaying the aging process of the fruit. This provides a new perspective for alleviating the core browning of advanced-maturity ‘Yali’ pears during storage and transportation, and provides a theoretical reference for studying the mechanism of core browning of ‘Yali’ pears.

## 1. Introduction

The ‘Yali’ pear (*Pyrus bretschneideri* Rehd cv. Yali) is one cultivar of white pear, which has long been a staple in Hebei Province, China. As the primary exported pear fruit, it is well-liked by customers [1,2]. When stored and transported properly, the ‘Yali’ pear can be kept until February or March of the next year after being harvested in September [3].

Core browning of the pear, which has been documented in cases of ‘Yali pears’, ‘Nanguo pear’, ‘Whangkeumbae pear’, ‘Bartlett’, ‘Korla pears’, and other pear kinds, is a common physiological disorder [4,5,6,7,8]. According to studies, the core browning of the ‘Yali’ pear occurs in a pattern that starts in the core of the fruit and then spreads to the flesh [3]. Insights into the mechanism of fruit browning have been studied extensively. The Vc protection concept, the regionalized distribution of phenols and phenol oxidase, and the oxygen radical theory are the three primary hypotheses [1,9]. The most extensively researched theory is the regionalized distribution theory, which argues that the core browning of the ‘Yali’ pear during storage is attributed to damage to the membrane function, interfering with the compartmentalization of metabolism [1]. Moreover, there are some reports arguing that the core browning of the ‘Yali’ pear was related to the activity of enzymes. For example, hypobaric treatment has the potential to manage core browning of the ‘Yali’ pear by regulating enzymes such as polyphenol oxidase (PPO) activity, succinate dehydrogenase (SDH), and glucose-6-phosphate dehydrogenase (G-6-PDH) [10]. The results of Liu et al. also indicated that the suppressive impact of fumigation with 20 µL of L^−1^ NO on core browning was linked to its ability to reduce PPO activity [11]. Atmosphere packaging with a 10 μm thickness reduced the core browning of ‘Yali’ pears and delayed the peak appearance of PPO activity [1]. The core browning under various cooling methods was found to be linked to the actions of superoxide dismutase (SOD), catalase (CAT), peroxidase (POD), glutathione reductase (GR), and lipoxygenase (LOX). These results suggest that PPO, SOD, SDH, CAT, POD, et al., might be involved in the core browning of ‘Yali’ pears.

Pear, as a typical respiratory climacteric fruit, undergo an after ripening process to achieve the optimal edible quality [12]. A variety of fruits can release ethylene, a gaseous hormone that is crucial for the ripening and senescence of respiratory climacteric fruits [13]. As ethylene inhibitors, 1-MCP and aminoethoxyvinylglycine (AVG) dramatically reduced browning of pear fruit after application. This suggests that by delaying the senescence of pear fruit, 1-MCP and AVG can prevent the development of browning in pear fruit [14].

The time of harvest, fruit quality, storage temperature, CO_2_ concentration, storage method, and exogenous preservative treatment are numerous variables that contribute to core browning in ‘Yali’ pears [15], and make it more susceptible to temperature changes; ‘Yali’ pears can be stored at 2–3 °C for 12 weeks and 0.5 °C (0.5 °C) for 30 weeks [3]. Our previous research demonstrated that the core browning of ‘Yali’ pears at different maturity levels can be alleviated by various cooling methods [3,16]. Therefore, we consider various cooling techniques for managing ‘Yali’ pears at advanced ripeness, assess the most effective cooling method for ‘Yali’ pears at advanced maturity, pinpoint physiological indicators related to the quality of ‘Yali’ pears and the percentage of good fruit, and utilize RNA-seq technology to investigate the impact of the cooling method on ethylene-related pathways of ‘Yali’ pears at the transcriptional level. This is to elucidate the mechanisms through which different cooling methods regulate the core browning of ‘Yali’ fruits at advanced maturity.

## 2. Materials and Methods

### 2.1. Fruit and Treatments

#### 2.1.1. Fruit

The ‘Yali’ pears in this experiment were harvested from Xinji City, Hebei Province, China. After careful selection, fruits with similar size, free from mechanical injuries, diseases, and pests were transported in cartons to the cold store of Tianjin Agricultural University. A total of 20 boxes, each containing 70 fruits were used, and there were two sets of 10 boxes for each cooling treatment.

#### 2.1.2. Cooling Treatment and Sampling

‘Yali’ pears were divided into two treatments: rapid cooling and slow cooling. In the rapid cooling treatment, the pears were pre-cooled at 0 °C for 24 h and then directly placed into cold store which maintained 0 ± 1 °C; for the slow cooling treatment, the pears were pre-cooled at 12 °C for 24 h and then placed into a 12 °C cold store, where the temperature was gradually reduced to 0 ± 1 °C in 30 days, by dropping 2 °C over a period of 5 days. Physiological indexes were measured every 30 days for the samples. The core parts of the pear were snapped frozen in liquid nitrogen and stored in a −80 °C refrigerator for subsequent experiments.

### 2.2. Core Browning Index of ‘Yali’ Pears

The core browning index of ‘Yali’ pears was determined by the method of Yan Shijie et al. [3]. In each storage time, 30 pears were randomly chosen from each treatment (10 boxes), and taken out of the cold store. These were then divided into 3 repetitions, with 10 pears in each repetition. The core browning of ‘Yali’ pears was observed and recorded at intervals of 0, 30, 60, 90, 120, 150, 180, and 210 days of storage. All the fruits were cut horizontally, and the degree of core browning was assessed based on the level of browning in the cross-section and the extent of browning in the core or flesh tissue. No browning was rated as 0; slight browning with brown spots in the core was rated as 1; slight to 20% browning was rated as 2; 21–50% browning was rated as 3; and greater than 50% browning was rated as 4. Subsequently, the core browning index was calculated according to the following formula:

$$Core\;browning\;index=\frac{\sum \left(Browning\;level\times The\;number\;of\;fruits\;of\;this\;level\right)}{The\;highest\;browning\;level\times total\;number\;of\;fruits}$$

### 2.3. Physiological Indexes Assessments

#### 2.3.1. Respiratory Intensity of Fruit

The measurement of fruit respiration intensity followed the method described by Li Ling et al. [16] with some modification. The respiratory intensity and ethylene release of ‘Yali’ pears were measured using fixed fruit and measured in cold store. For the selection of fixed fruits, 12 fruits were randomly selected, with 4 fruits in each group, and a total of 3 groups before storage. Next, the grouped pears were placed in a modified Lekou box, sealed for 1 h, and the gas inside the box was extracted using a 1 mL medical syringe. A total of 4 needles were extracted from each box, with 3 for repetition and 1 left as a backup. The respiration intensity of the ‘Yali’ pears was measured using a GXH-3051 infrared CO_2_ analyzer (Beijing Junfang Physics & Chemical Science & Technology Research Institute, Beijing, China), and calculated using the calculation formula as follows (the unit was CO_2_ mg kg^−1^ h^−1^):

$$Fruit\;respiration\;intensity=\frac{F\times 60\times C}{22.4}\times \frac{44}{W}\times 10^{-6}\times \frac{273}{273+T}$$

in which F is the flow rate of gas, the unit was expressed in mL min^−1^;
C is the CO_2_ concentration, the unit was expressed in μL L^−1^;
W is the weight of the measured fruit, the unit was expressed in kg; and
T is the temperature at the time of measurement, the unit was expressed in °C.

#### 2.3.2. Respiratory Intensity of Seed

For each treatment, a total of 30 relatively full seeds were selected and divided into 3 groups. During the storage period, the seeds were placed in 10 mL glass vials with rubber caps, which were then sealed for 1 h, and 0.5 mL of gas was extracted from each vial. The calculation of respiratory intensity was the same as Section 2.3.1.

#### 2.3.3. CO_2_ Content of Core

A total of 10 ‘Yali’ pears were selected for the experiment. By using a syringe, the core chamber of ‘Yali’ pear was pierced from the center part of the calyx, then 0.5 mL of gas was extracted for analysis, following the same method as described in Section 2.3.1, and the unit was μL L^−1^.

#### 2.3.4. Ethylene Production

The ethylene gas was obtained in the same way as the respiratory intensity and was measured by GC-14 gas chromatograph (Shimadzu, Kyoto, Japan) and repeated three times. The setup parameters and calculation formula were referred from Li Ling et al. [16] with some modification. The experimental parameters were set as follows: the chromatographic column was a GDX-502 stainless steel packed column; the detector used was a hydrogen flame ionization detector; and the carrier gas was N_2_. The inlet temperature, column temperature box, and detector temperature were all set at 60 °C, and the unit was expressed in μL kg^−1^·h^−1^.

$$Ethylene\;Production=\frac{C\times V}{M\times T\times 1000}$$

in which C is the ethylene content in the sample gas, with the unit as µL L^−1^;
V is the volume of the enclosed space, with the unit as mL;
M is the mass of fruits, with the unit as kg; and
T is the smothering time, with the unit as h.

#### 2.3.5. Soluble Solids Content and Titratable Acids

Refer to the method of Li Jian et al. [7] with some modification. At each storage time for testing, 15 fruits were randomly selected from each treatment (10 boxes), taken out of the cold store, and divided into 3 groups. Fruits were peeled and cut to extract juice from the pulp part, and the soluble solids content (SSC) of the juice was measured by PAL-10 (Atago, Tokyo, Japan). The titratable acid (TA) was measured by GMK-835N acidimeter (Beijing Shangdetong Technology Co., Ltd., Beijing, China).

#### 2.3.6. Firmness

The firmness of ‘Yali’ pears were determined by referring to the method of Li Jian et al. [7]. At each storage time for testing, 15 fruits were randomly selected from each treatment (10 boxes), taken out of the cold store, and divided into 3 groups. The firmness of ‘Yali’ pear fruits were determined using a TA-XT plus physical property analyzer (Stable Micro System, Godalming, UK). The unit was expressed in kg cm^−2^.

#### 2.3.7. L*, a* and b* Value

The color of ‘Yali’ pears was determined with reference to the method of Li Jian et al. [7]. For the determination of L*, a* and b* values, 15 fixed ‘Yali’ pears were used and tested in cold store, and divided into 3 groups. The fruits were marked in the middle with a circle of approximately 2.0 cm in diameter. Measurements were carried out using a SC-10 colorimeter (Kunshan Henggang Electronic Technology Co., Suzhou, China) to record the values of L*, a* and b* of the ‘Yali’ pear fruit during storage.

#### 2.3.8. Good Fruit Rate

After 210 days of storage, the remaining ‘Yali’ pears were counted to determine the rate of good fruit.

$$Good\;fruit\;rate=\frac{Number\;of\;good\;fruits\;per\;treatment}{total\;number\;of\;statistics\;per\;treatment}$$

### 2.4. RNA-Seq

The core of ‘Yali’ pears was sampled at 0 days and 60 days after slow cooling and rapid cooling treatment. Total RNA was extracted using the Trizol reagent kit following the manufacturer’s protocol. The quality of RNA extraction was detected by 1% agarose gel electrophoresis, and the concentration and purity of RNA were detected by NanoDrop 2000 spectrophotometer (Thermo, Waltham, MA, USA). Subsequently, mRNA was enriched with Oligo (dT) beads. The enriched mRNA was then fragmented into short fragments using fragmentation buffer and reverse transcribed into cDNA with random primers. Second strand cDNA synthesis was carried out using DNA polymerase I, RNase H, dNTPs, and buffer. The cDNA fragments were purified with the QiaQuick PCR extraction kit, end-repaired, poly(A)-tailed, and ligated to Illumina sequencing adapters. The ligation products were size-selected via agarose gel electrophoresis, PCR amplified, and sequenced using Illumina HiSeq2500 (Illumina, San Diego, CA, USA) by Beijing Novogene (Beijing, China).

### 2.5. Determination of ACO Activity

The determination of ACC oxidase (ACO) activity was referred from Fonseca et al. [17] with some modification. The sample from the core of a ‘Yali’ pear was taken, and enzyme extract and enzyme activity determination and computing method were carried out as in references. After the experiment, 1 mL of gas was extracted from the headspace, and the ethylene release was measured by Shimadsu GC-14. The ACO activity was expressed as the production rate of ethylene per hour, and the ACO activity was expressed as nmol h^−1^g^−1^.

### 2.6. Statistical Analysis

Each trial was executed with three independent biological duplicates. Data reorganization and standard errors were computed using Excel 2019. All statistical assessments were carried out utilizing SPSS 22 (IBM Inc., Armonk, NY, USA). Differences between the groups on a single storage day were appraised through *t*-test. Mean separations were determined using the least significant difference (LSD) test. A *p*-value of 0.05 was established as the threshold for significance. Figures were generated using GraphPad Prism 8.

## 3. Results

### 3.1. Development Process of Browning

**Figure 1:**
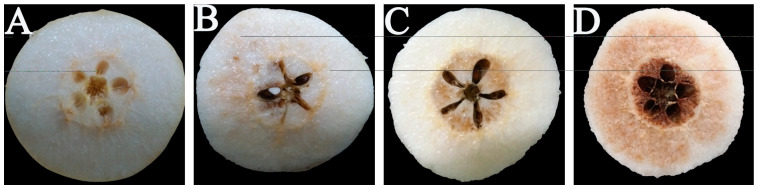
Development process of core browning in ‘Yali’ pears. (**A**): the core of ‘Yali’ pears completely in a non-browning state; (**B**): the initial browning in the locule; (**C**): the core of ‘Yali’ pears completely browned, (**D**): the browning extended from the core to the pulp.

Figure 1A shows the core of ‘Yali’ pears completely in a non-browning state; Figure 1B illustrates the initial browning in the locule; Figure 1C shows the core of ‘Yali’ pears completely browned, and Figure 1D shows the browning extended from the core to the pulp. The progression from Figure 1A–D represented the development of fruit browning. It was evident that the browning of ‘Yali’ pears originally started from the core chamber, and gradually spread to the pulp.

### 3.2. Effects of Different Cooling Treatments on Browning Index of ‘Yali’ Pears at Advanced Maturity

**Figure 2:**
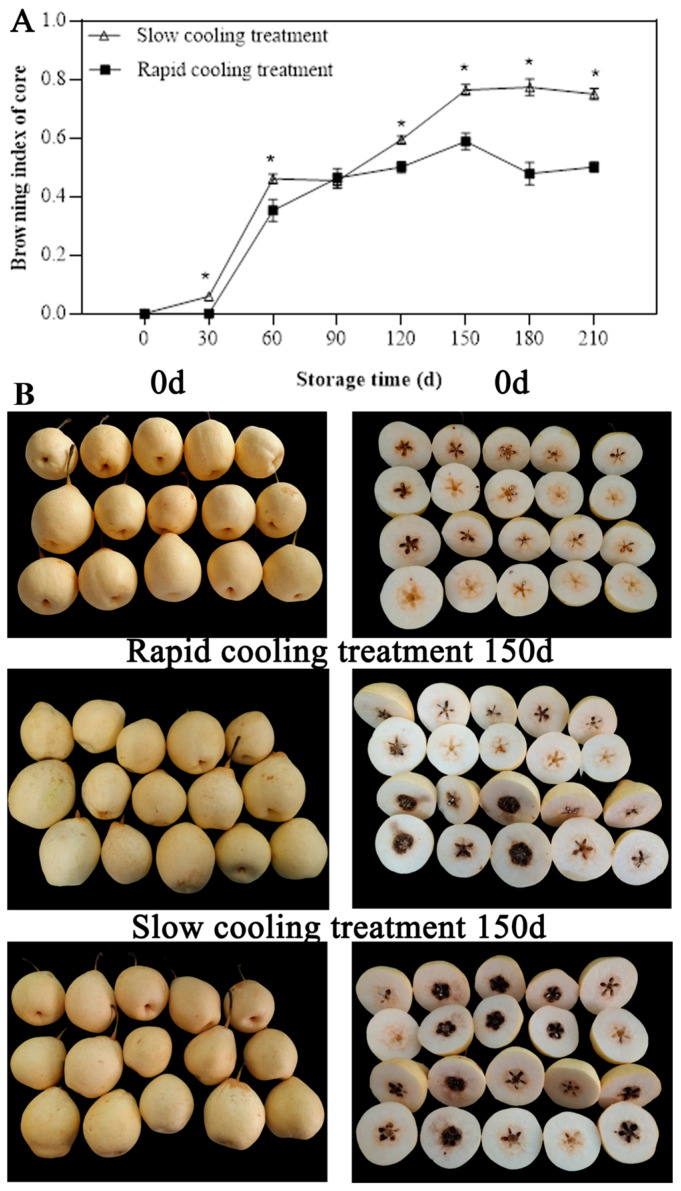
Core browning index (**A**) and the browning phenotype (**B**) of ‘Yali’ pears. Each value in (**A**) is the mean ± SE, *n* = 3. Asterisks (*) indicate significant differences between the two fruit treatments (*p* < 0.05).

As shown in Figure 2A, the browning index of ‘Yali’ pears increases gradually as the storage period extended. The occurrence time of browning in ‘Yali’ pears differed between the two cooling treatments. With the slow cooling treatment, slight browning was observed at 30 days, while it occurred at 60 days with the rapid cooling treatment. During the storage period of 60 to 150 days, there was a sharp increase in the browning index. Notably, the browning was more pronounced in the slow cooling treatment. Towards the end of storage, the browning index of the slow cooling treatment exceeded that of the rapid cooling treatment, with the rapid cooling treatment exhibiting a browning index of 0.5 and the slow cooling treatment reaching as high as 0.75. Figure 2B depicts the browning state of ‘Yali’ pears after 210 days of storage.

### 3.3. Effects of Different Cooling Treatments on Physiological Indexes of ‘Yali’ Pears at Advanced Maturity

**Figure 3:**
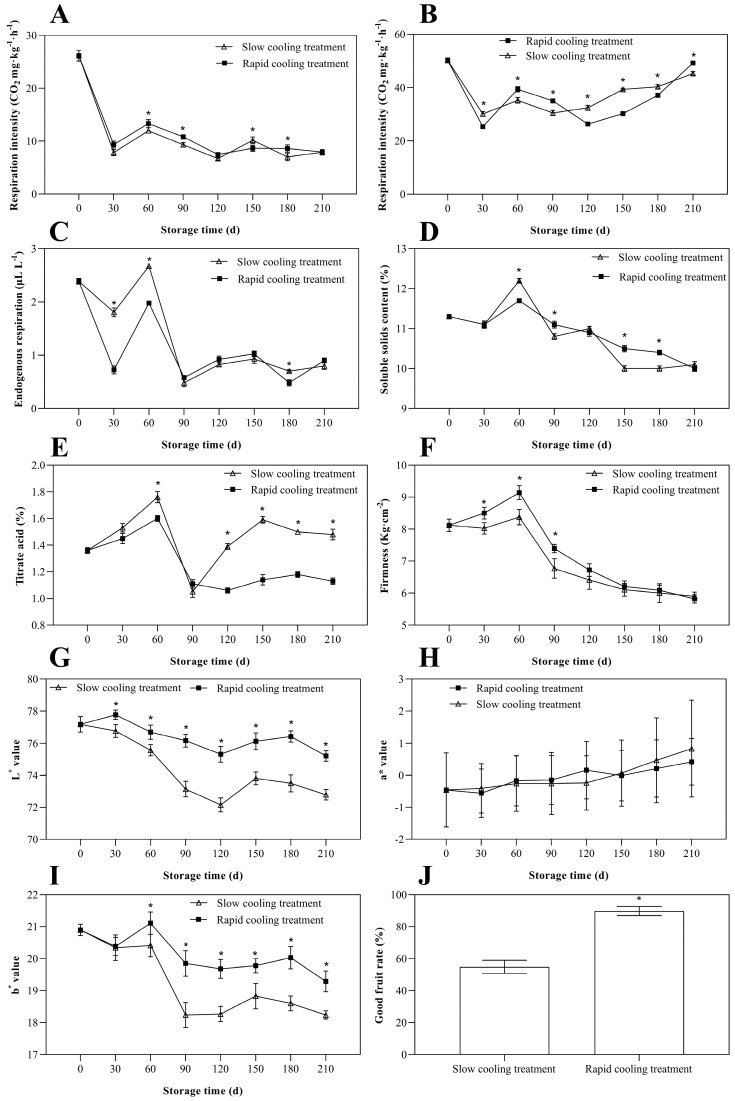
Physiological indexes of ‘Yali’ pears: the respiratory intensity of fruit (**A**), the respiratory intensity of seed (**B**), endogenous respiration (**C**), soluble solids content (**D**), titratable acids (**E**), firmness (**F**), L* value (**G**), a* value (**H**), b* value (**I**), and the good fruit rate (**J**). Each value is the mean ± SE, *n* = 3. Asterisks (*) indicate significant differences between the two treatments.

As shown in Figure 3A, the respiratory intensity of freshly harvested ‘Yali’ pears was 26.48 mg CO_2_ kg^−1^h^−1^. With the extension of storage duration, the respiration intensity initially decreased and then exhibited an upward trajectory, reaching its peak at 60 days. In general, the respiration intensity of the rapid cooling treatment surpassed that of the slow cooling treatment at 30, 60, 90, 120, and 180 days of storage.

Figure 3B illustrates the changes in the respiratory intensity of seeds. Initially, the respiratory intensity of seeds was high when the fruit was freshly harvested. With the extension of the storage, the respiratory intensity first decreased and then peaked, and subsequently decreased again. Towards the end of the storage, there was a tendency for an increase. The respiratory intensity of slow cooling treatment was higher than that of rapid cooling treatment at 30 days and 120–180 days.

The CO_2_ accumulation in the core showed a similar pattern as the respiration intensity in fruits. As demonstrated in Figure 3C, the CO_2_ accumulation in the core first decreases, then increases to a peak, followed by another decrease. The CO_2_ accumulation in the core peaked at 60 days, which aligned with the results of respiration intensity in seeds as well as in fruits. The CO_2_ accumulation was slightly higher in the slow cooling treatment than in the rapid cooling treatment.

Figure 3D depicts that as storage time increases, the SSC first increases to the maximum value and then declines. Towards the end of storage (150~180 days), SSC decreased rapidly, with the SSC of the slow cooling treatment being lower than that of the rapid cooling treatment.

As shown in Figure 3E, the TA reaches the peak at 60 days and then decreases as the storage extends, and finally plateaus. Up to the middle of storage (60~90 days), the content of titratable acid decreased sharply, and in the late storage period (120–210 days), the TA of the slow cooling treatment was greater than that of the rapid cooling treatment.

The initial firmness of ‘Yali’ pears was 8.12 kg cm^−2^ as shown in Figure 3F, with the increasing storage time, the firmness initially peaks at 60 days, then decreases and finally plateaus. At the beginning of storage (30–120 days), rapid cooling significantly kept the firmness of ‘Yali’ pears, and towards the end of storage (120–210 days), there was no difference between the firmness of the slow cooling treatment and that of the rapid cooling treatment.

L* indicates the degree of brightness of ‘Yali’ pears, and the larger the L*, the brighter the color of the peel. Figure 3G reveals that the L* value of the ‘Yali’ pear after picking decreases, and the L* value of the slow cooling treatment is significantly lower than that of the rapid cooling treatment.

The a* value represents the redness (+a*) or greenness (−a*) of a color. The a*-values of both treatments showed an increase with the prolongation of storage time, and no notable discrepancy in the a-values was observed between the two treatments at each measurement time point (Figure 3H).

Figure 3I presents the changing of b* values. A b* value indicates the change between yellow (+b*) and blue (−b*), which decreases with extended time in the storage, and b* values of the slow cooling treatment were lower than that of the rapid cooling treatment.

As shown in Figure 3J, good fruit rate wis 89.76% in the rapid cooling treatment group, and 54.84% in the slow cooling treatment group. This indicated that the rapid cooling treatment was better for ‘Yali’ pears at advanced maturity.

### 3.4. GO and KEGG Enrich Analysis of DEGs

**Figure 4:**
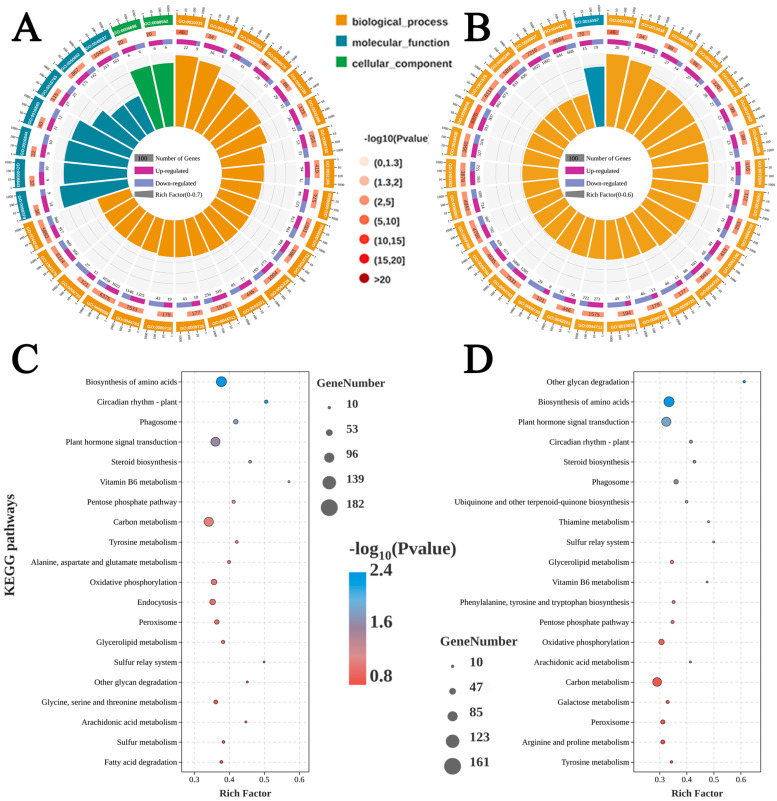
The GO enrichment analysis of DEGs from 0 days and 60 days after slow cooling treatment (**A**); 0 days and 60 days after rapid cooling treatment (**B**); the KEGG enrichment analysis of DEGs from 0 days and 60 days after slow cooling treatment (**C**); and 0 days and 60 days after rapid cooling treatment (**D**). *p*-value: statistically significant level of enrichment analysis; the color represents the level of *p*-value, where the larger the −log_10_ (*p* value), the smaller the *p*-value, indicating that the pathway is more significant.

Figure 4 illustrates the GO and KEGG enrichment analysis of all differentially expressed genes (DEGs) from core samples of ‘Yali’ pear treated with various cooling methods for 60 days compared to 0 days. The subclasses of response to metal ion, response to endogenous stimulus, and cellular amino acid metabolic process were connected to the pear’s stress response to the environment; other subcategories included amino acid metabolism, and other life activities, according to GO enrichment analyses (Figure 4A,B). The top 20 primary important KEGG pathways were presented, with amino acid biosynthesis, plant hormone signal transduction, and carbon metabolism ranking as the top three, which were found to be linked to resistance and senescence in the ‘Yali’ pear (Figure 4C,D).

### 3.5. Ethylene Metabolism in ‘Yali’ Pears during Slow Cooling and Rapid Cooling Treatment

**Figure 5:**
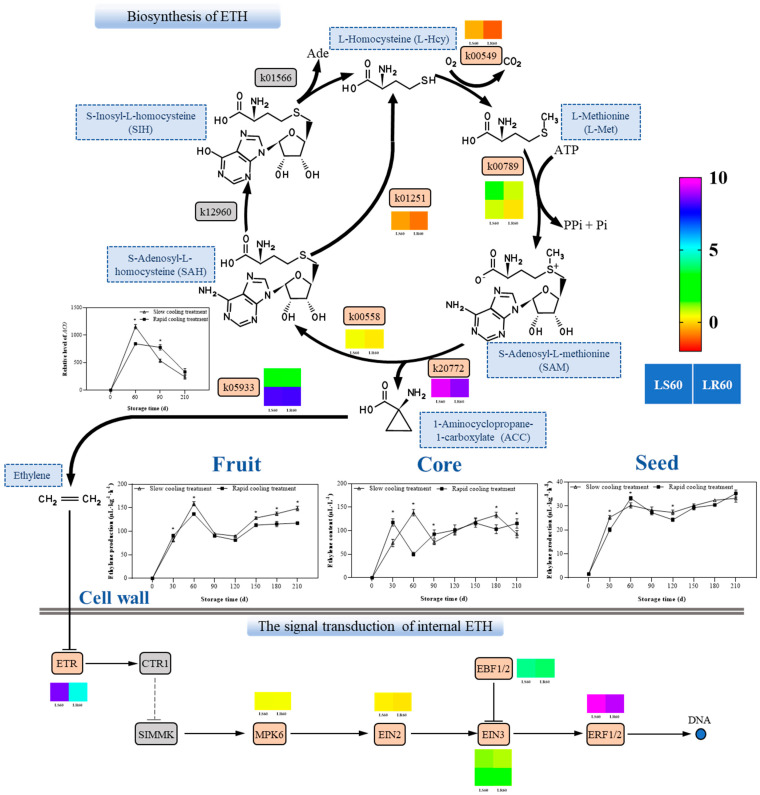
Biosynthesis and signal transduction of internal ETH in ‘Yali’ pears after the slow cooling and rapid cooling treatment at 60 days. Note: The heat map next to the gene in the figure represents the FPKM of the gene in the slow cooling and rapid cooling treatment at 60 days. K00789: S-adenosylmethionine synthetase, SAMS; K00558: DNA (cytosine-5)-methyltransferase 1, DNMT1; K20772: 1-aminocyclopropane-1-carboxylate synthase, ACS; K12960: 5-methylthioadenosine/S-adenosylhomocysteine deaminase, mtaD; K01251: aminocyclopropanecarboxylate oxidase, AHCY; K01566: S-inosyl-L-homocysteine hydrolase, SIHH; K00549: 5-methyltetrahydropteroyltriglutamate—homocysteine methyltransferase, metE; and K05933: aminocyclopropanecarboxylate oxidase, ACO. Each value is the mean ± SE, *n* = 3. Asterisks (*) indicate significant differences between the two treatments.

Figure 5 shows the biosynthesis and signal transduction of internal ETH in ‘Yali’ pears after slow cooling and rapid cooling treatment at 60 days. The expression levels of genes related to ethylene biosynthesis (*SAMS*, *ACS*, *ACO*) as well as genes related to ethylene signal transduction pathway (*ETR*, *MPK6*, *EIN2*, *EIN3*, *EBF1/2* and *ERF1/2*) were higher in ‘Yali’ pear treated with the slow cooling treatment compared to those treated with the rapid cooling treatment. Additionally, the fruit and core exhibited extremely low ethylene release in the rapid cooling treatment—nearly zero. As storage time was extended, the ethylene release of fruit, core, and seed gradually rose until it reached its peak at 60 days, and then declined before showing a tendency to increase again. In general, the ethylene release of fruit was higher in ‘Yali’ pear treated with the slow cooling treatment compared to those treated with the rapid cooling treatment, which was consist with the expression of related genes.

## 4. Discussion

Core browning reduces the commodity value of the fruit and shortens its market supply time and sales [7]. The browning process in ‘Yali’ pear fruits progress through multiple stages, from the core to the flesh (Figure 1). During the observations, we classified the fruits based on the degree of browning, which ultimately determined the browning index [3]. According to our research, the core browning signs in advanced-maturity ‘Yali’ pears appeared after 30 days when treated with the slow cooling method, and at 60 days when treated with the rapid cooling method. As storage was prolonged, the core browning index of ‘Yali’ pears treated with the rapid cooling were significantly lower than that of the slow cooling treatment group (Figure 2), indicating that the rapid cooling treatment can inhibit core browning incidence and achieve a higher rate of good fruits for advanced-maturity pear fruits.

Respiratory intensity and ethylene production indicate the plant’s vitality and are closely linked to the quality and senescence process of pears during storage [18]. The cooling methods have an effect on the respiratory intensity of ‘Yali’ pear fruits. In the early storage period (0–90 days), the respiration intensity of the rapid cooling treatment group is higher than that of the slow cooling treatment group, indicating that rapid cooling promoted physiological metabolism of the ‘Yali’ pear. In the late storage period (150–210 days), there is almost no difference in respiration intensity between the slow cooling and rapid cooling groups (Figure 3A). The regularity in respiration intensity of seed is consistent with the results of fruit, indicating that the internal physiological metabolism of seeds affects the physiological metabolism of fruits. At the late stage, as fruits tend to senesce and seeds begin to germinate, there is an increase in respiration intensity (Figure 3B). There are numerous variables that contribute to core browning in the ‘Yali’ pear, with CO_2_ concentration being one of them [2,3]. We examined CO_2_ accumulation in the core using the pinprick method to investigate the correlation between CO_2_ accumulation in the core and core browning in ‘Yali’ pears. The peak of CO_2_ buildup occurred at 60 days, which corresponded with the respiration intensity of both the fruit and the seeds. At the same time, ‘Yali’ pears exhibited varying degrees of core browning at 60 days due to the rapid accumulation of CO_2_ during the early storage period, resulting in damage to the core tissues. The core browning in the slow cooling treatment was higher than that in the rapid cooling treatment, likely due to slightly higher CO_2_ accumulation in the slow cooling treatment (Figure 3C).

High-quality fruits have been increasingly popular in recent years, and TA and SSC are two significant determinants of fruit quality [7]. SSC and TA changes could be attributed to the conversion of sugars and acids during fruit ripening [19]. The type and content of soluble sugars in fruits not only determine the flavor and quality of the fruit, but they are also important energy substances, intermediates, and important substrates of many metabolic pathways, and they play a role in the regulation of plant growth, ripening, and senescence as signaling molecules in many processes; for example, the delay of fruit senescence was related to the maintaining of higher energy status in apple fruit [20]. A higher amount of SSC content can prolong the storage life of ‘Yali’ pears and prevent core browning and senescence; our results also showed a correlation between core browning and SSC content, as SSC declined rapidly towards the end of the storage period (120–210 days), coinciding with the severe browning of fruit (Figure 3D). At 60 days, TA reached its peak (Figure 3E). This indicated that increased SSC might partially prevent core browning and delay TA degradation. Due to extensive browning in advanced-maturity ‘Yali’ pears, the firmness of the fruits was at its lowest at the end of the storage period (120–210 days), particularly with the slow cooling treatment, which led to more pronounced core browning. Consequently, the fruit firmness decreased significantly compared to the rapid cooling treatment group (Figure 3F). The brightness of ‘Yali’ pears also decreased more in the slow cooling treatment group than in the rapid cooling treatment group (Figure 3G). This was due to the prolonged storage time causing severe core browning. Furthermore, the brown index was higher for the slow cooling treatment group compared to the rapid cooling treatment group as it extended from cores to flesh, resulting in reduced peel brightness.

In order to further explore how the rapid cooling treatment alleviated the browning of advanced-maturity ‘Yali’ pears, we investigated the mechanisms by RNA-Seq sequencing, and the results showed that the GO enrichment related to browning mainly focused on biosynthetic process and molecular function (Figure 4), and the DEGs in GO term focused on the stimulation aspect, single cell process, and metal-binding activity. Regarding KEGG pathway annotation, genes causing browning were found in amino acid metabolism, plant hormone signal transduction, and carbon metabolism pathways. According to a report, maturity and senescence of ‘Nanguo’ pears are directly related to the core browning of the fruit at room temperature [6]. Advanced-maturity ‘Yali’ pears underwent various cooling treatments, resulting in browning caused by alterations in metabolic and activity levels of browning-related substances during the storage of ‘Yali’ pears, and we focused on DEGs associated with fruit senescence.

It is generally recognized that ripening and senescence serve as the causes of browning in ‘Yali’ pears [15]. One factor that accelerates the senescence of fruit and causes browning is the ethylene released during the storage of fruit [21]. Having reported that the respiratory intensity and ethylene production serve as indicators of the plant’s vigor and are intimately related to the quality and senescence process of pears during storage [18], any intervention that delays fruit senescence or prolongs the storage period of fruit can only postpone the peak of ethylene or decrease ethylene production. For instance, the low temperature controlled the ethylene release rate to a certain extent during an 80 day low-temperature storage, but the cold conditions did not change the general trend of increasing ethylene release in ‘Meihong’ fruits [22]. Furthermore, with the ripening of fruits at RT (25 ± 1 °C), ethylene release increased and peaked at 20 days; for fruits under the LR (2 ± 1 °C for 10 days then 25 ± 1 °C) environment, ethylene release remained almost unchanged during the first 10 days but peaked at 15 days [23]. ‘Yali’ pears are climacteric fruits with a peak ethylene release at around 60 days of storage, coinciding with their peak respiratory intensity (Figure 5). Throughout the late storage period (120–210 days), especially in the slow cooling treatment group, core browning of fruits became more severe as indicated by an increase in fruit browning index due to continuous ethylene release leading to nutrient consumption. The ethylene release of the slow cooling treatment group was higher than that of the rapid cooling, and at the same time, the browning level of slow cooling was higher than that of rapid cooling.

Ethylene, as a little compound, could regulate many metabolic and developmental processes in plants, including seed germination, seedling development, organ growth and senescence, fruit ripening, and stress response [18]. L-Met is the primary base substance for the synthesis of ethylene in plants. S-adenosylmethionine synthetase (SAM), 1-aminocyclopropane-1-carboxylic acid synthetase (ACS), and 1-aminocyclopropane-1-carboxylic acid oxidase (ACO) regulate this process in turn. Ethylene molecules are produced by the catalysis of three enzymes [13]. The *ACS* and *ACO* gene families are the two most significant genes involved in ethylene production out of these three genes. The alternating expression of the *ACO* and *ACS* genes regulate ethylene production during the developmental and ripening phases of climacteric fruit, completing the progression from development to ripening. The ‘Yali’ pear is a type of fruit known as a respiratory climacteric fruit. During the postharvest ripening process, there is an obvious accumulation of ethylene that motivates the organism to accelerate maturation. This is accompanied by several changes including an increased respiration rate in fruits, decreased flesh firmness, accumulation of soluble solids content, increase in flavor compounds content, etc.

In this experiment, the rapid cooling treatment dramatically reduced the ethylene release of ‘Yali’ pears compared to the slow cooling treatment group. Rapid cooling enables ‘Yali’ pears to adapt more quickly to a low temperature environment. Additionally, it reduces the metabolic rate of the fruit overall than slow cooling and releases less amounts of ethylene, which delays the core browning related to senescence. Thus, it has been established that rapid cooling prevented the development of core browning in ‘Yali’ pears at advanced maturity.

## 5. Conclusions

In our study, the application of rapid cooling resulted in a decrease in the browning index of ‘Yali’ pears. Additionally, the peaks in respiration for ‘Yali’ pear fruits, seeds, and core chambers treated with the two different cooling methods all occurred at 60 days during the storage. Overall, the fruits treated with slow cooling exhibited higher respiratory intensity and ethylene production compared to those treated with rapid cooling. Moreover, the rapid cooling group showed higher levels of soluble solids, firmness, L-value, b-value, and a higher rate of good fruits, as compared to the slow cooling treatment group. These findings suggest that for advanced-maturity ‘Yali’ pears, rapid cooling can reduce ethylene production and respiratory intensity, thereby delaying the senescence process and preserving fruit quality. Consequently, this approach could lead to a decrease in the core browning index of the fruit, offering potential benefits for commercialization.

## Data Availability

The original contributions presented in the study are included in the article, further inquiries can be directed to the corresponding author.
